# Loss of Klotho contributes to cartilage damage by derepression of canonical Wnt/β-catenin signaling in osteoarthritis mice

**DOI:** 10.18632/aging.102603

**Published:** 2019-12-30

**Authors:** Yanqing Gu, Kewei Ren, Liming Wang, Qingqiang Yao

**Affiliations:** 1 Department of Orthopedics, Nanjing First Hospital, Nanjing Medical University, Nanjing, Jiangsu, China; 2 Department of Orthopedics, The Affiliated Jiangyin Hospital of Medical School of Southeast University, Jiangyin, China; 3 Cartilage Regeneration Center, Nanjing First Hospital, Nanjing Medical University, Nanjing, Jiangsu, China; 4 Digital Medicine Institute, Nanjing Medical University, Nanjing, Jiangsu, China

**Keywords:** Klotho, osteoarthritis, WNT, MMP

## Abstract

Caducity is known to be an independent risk factor in osteoarthritis (OA), yet the molecular basis behind caducity and OA remains unclear. Klotho, an anti-caducity protein, is an endogenous antagonist of the transduction of Wnt/β-catenin signal which can stimulate the articular cartilage degradation, indicating that deficiency in Klotho may increase Wnt/β-catenin activity and consequently accelerate the development of OA. We found that expression of Klotho was markedly higher in normal mouse cartilage than in the OA model, and in this model the activity of Wnt/β-catenin and its target gene was up-regulated. Decrease in Klotho expression was closely associated with the increase of β-catenin in OA, indicating that there was a negative correlation between Klotho and Wnt signal transduction. In the vitro and in vivo experiments, Klotho was found to bind to multiple Wnt, including Wnt1, Wnt4 and Wnt7a. It was additionally found that cyclic tenisle strain (CTS) inhibited the expression of Klotho and activated β-catenin. On the contrary, over-expression of Klotho would reduce the degradation of articular cartilage induced by CTS. These results suggest that Klotho is an antagonist of endogenous Wnt/β-catenin activity. In OA cartilage, decrease in expression of Klotho can activate Wnt/β-catenin signal transduction and consequently induce cartilage injury.

## INTRODUCTION

Osteoarthritis (OA), characterized by sub-chondral synovial activation, bone remodeling, and progressive loss of cartilage homeostasis, is generally regarded as the primary age relevant osteoarticular disease [1, 2]. The molecular mechanism underlying OA was recently deciphered [2]. Articular chondrocyte acquisition of a p16INK4A-dependent senescence phenotype may be induced by repetitive mechanical stress as well as its consequent synovial inflammation [3], which is virtually identical with the terminal differentiation during endochondral ossification [3, 4].

Klotho, named after a Greek goddess Klotho, was identified as a gene mutated in Klotho mice in 1997, which is primarily expressed in reproductive organs, kidney and brain [5, 6]. Mice homozygous for the transgene have a number of phenotypes similar to the premature aging syndrome, such as calcification, pulmonary emphysema, osteoporosis, short life span and skin atrophy. This study verified the expression of Klotho in cartilage and confirmed the down-regulation of Klotho in the tissue of OA models, as shown in previous studies [7]. The association between Klotho and osteoarthritis has been already verified in several studies. Anti-geronic secreted Klotho can not only protect the articular cartilage, and additionally its repression with OA onset is also closely correlated. In addition, OA onset can also be predisposed by two single nucleotide polymorphisms inα-Klotho, i.e. genes that encode the anti-geronic hormoneα-Klotho [8–10].

The mechanism underlying the protection of cartilage from Klotho still remains unclear. Previous studies suggest that in renal injury model, Klotho expressed in renal tubular epithelial cells can bind to Wnt1, 4 and 7a to form a complex, which inhibits the activation of Frizzled and LRP5/6 complex, and plays a similar role to Wnt1, 4 and 7a antagonist. In this study, we hypothesized that Klotho would play a similar role in chondrocytes [11].

In the pathogenesis of OA, the activation of classical Wnt signal transduction is observed in articular cartilage and injured synovium, and the expression of Wnt ligand and target gene increases [12–17]. The expression of matrix metalloproteinase (MMPs) gene is regulated by Wnt/β-catenin signal transduction pathway. In the transgenic mice, activated β-catenin increases the expression of MMP-2, MMP-9 and MMP-13 [15, 18]. The MMPs is the primary enzyme that destroys the extracellular matrix (ECM) of articular chondrocytes. The over-expression of β-catenin in mature chondrocytes may aggravate the cartilage degeneration, increase chondrocyte hypertrophy and increase the MMPs expression [19–21].

Our experiment verified that low expression of Klotho in chondrocytes in OA also lost its antagonistic effect on Wnt1, 4 and 7a, which resulted in relative activation of Wnt/β-catenin and high expression of target gene MMP-2 and MMP-9. Finally, the degradation of the cartilage matrix was caused.

The activation of Wnt/β-catenin signal transduction in articular chondrocytes leads to chondrocyte hypertrophy and ECM degradation. Klotho regulation of Wnt/β-catenin signal may improve the development of OA. This study aims to determine whether the reduction of Klotho expression in OA may induce the over-expression of Wnt/β-catenin signal and the degradation of cartilage matrix, and whether Klotho is a key factor in the pathogenesis of OA under this condition.

## RESULTS

### HE staining and acin blue staining findings

**Figure 1:**
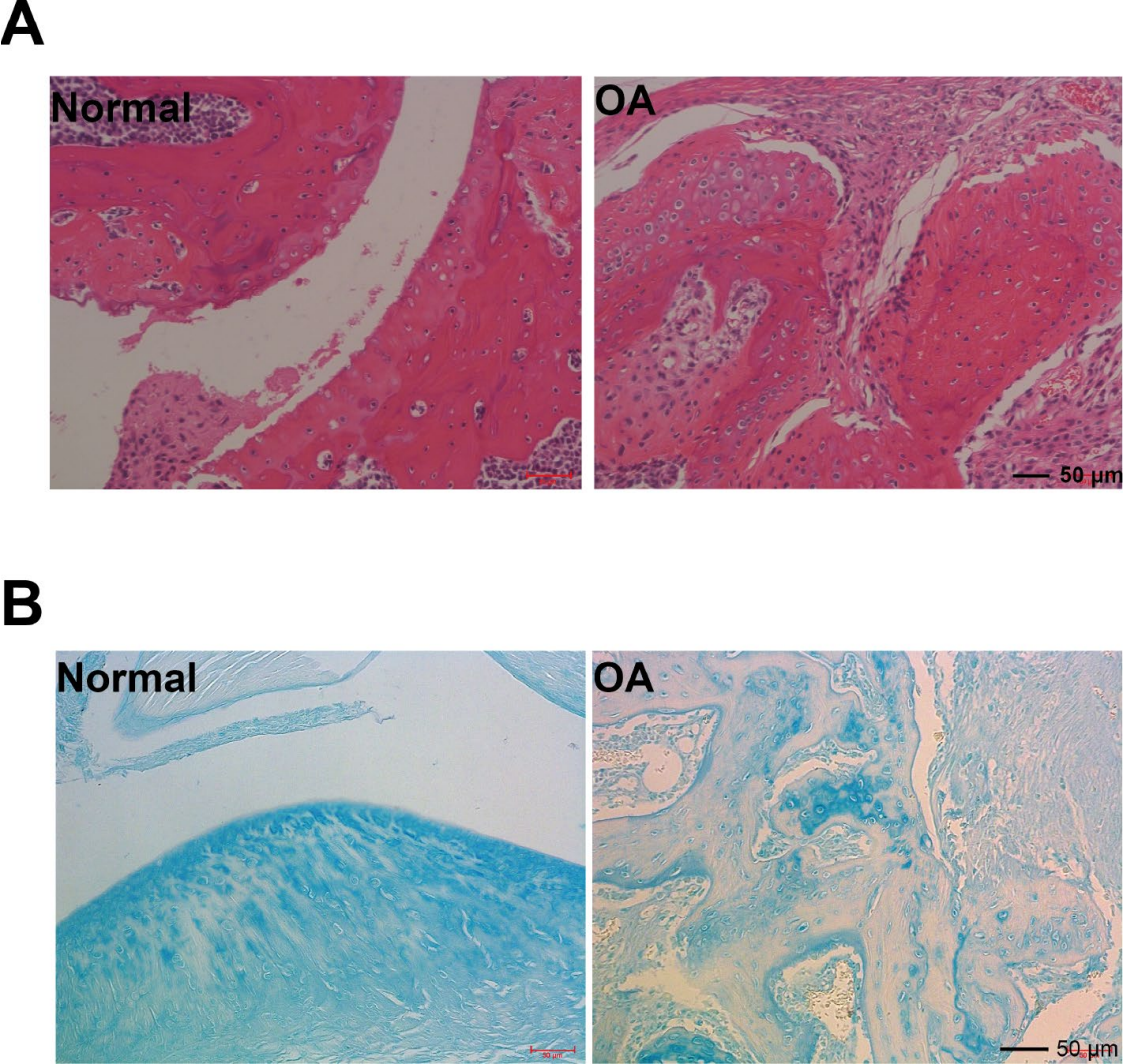
**Hmatoxylin and eosin (H&E), and acin blue staining.** (**A**) Representative photomicrographs HE staining of joint pathological sections of normal mouse joints and OA model mice, twelve weeks after ACLT (n=4). (**B**) Alcian blue staining of normal mouse joints and surgically prepared OA model mouse joint pathological sections (n=4).

Findings of representative photomicrographs HE staining and acin blue staining of joint pathological sections of normal mouse joints and OA model mice were available twelve weeks after ACLT. Intact and smooth cartilages were showed on sham-operated knee joints (Figure 1A, 1B). Additionally, a typical healthy cartilage was presented in the chondrocyte organization with flat cells in the superficial zone (one or two layers). In the middle and deep zones, columns of round cells were seen. In contrast, in male C57/6J mice, cartilage damage and disorganized chondrocyte were presented in the operated knees (ACLT).

### Expression of Klotho, wnt/β-catenin signal pathway and its target gene in the OA model

**Figure 2:**
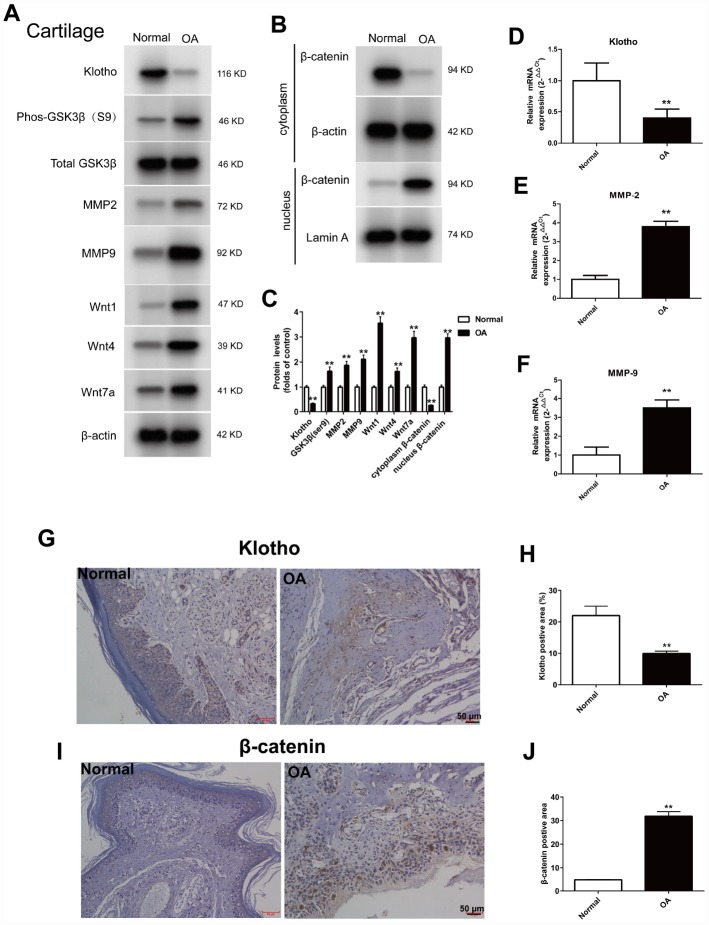
**Up-regulation of Wnt signaling and down-regulation of Klotho in the joint after injury.** ACLT surgery was used to induce OA in adult male C57/6J mice. Expression of Klotho, Phos-GSK3β (S9), GSK3β, MMP2, MMP9, Wnt1, Wnt4, Wnt7a and β-catenin (cytoplasm/nucleus) in the mouse cartilage tissue. (**A**) Expression change of Klotho, Phos-GSK3β (S9), GSK3β, MMP2, MMP9, Wnt1, Wnt4, Wnt7a, and (**B**) β-catenin in the cytoplasm and nucleus by Western Blot after modeling. (**C**) Protein levels of Klotho, Phos-GSK3β (S9), GSK3β, MMP2, MMP9, Wnt1, Wnt4, Wnt7a and β-catenin were detected by western blotting. Protein/β-actin and Protein/Lamin A were used to demonstrate the protein fold changes. (**D**) Klotho mRNA expression was assessed in the cartilage at 12 weeks after ACLT or control. (**F**) The expression of matrix-degrading enzyme genes, MMP-2 (**E**), MMP-9 (**F**) were evaluated by Real-Time PCR. (**G**–**J**) The expression of Klotho protein and β-catenin protein in the control group and OA group were compared by immunohistochemistry,. Scale bar, 50 mm. Data are the mean ±SD, n=8 mice. *P < 0.05, **P < 0.01.

To further examine the expression of Klotho in OA, a mouse model was established. Cartilage tissue was removed from the knee joint with anterior cruciate ligament transection, and then the expression of Klotho in articular cartilage of OA mice was observed. The results showed that the level of Klotho in OA mice decreased markedly (Figure 2A, 2D) after operation, and the immunostaining intensity of Klotho in the articular cartilage of OA mice decreased (Figure 2G).

On the contrary, the increased expression of Wnt signaling pathway and its target gene MMP2 and MMP9 in the cartilage of OA mice, and the increased expression of Phos-GSK3β (S9), Wnt1, Wnt4 and Wnt7a were observed by western blot, indicating that the Wnt signaling pathway was activated in OA (Figure 2A).

Wnt/β-catenin signal transduction pathway regulates the expression of MMPs gene downstream [18, 19]. MMPs are the primary enzymes that damage the ECM of the articular cartilage. Our results confirmed that, after activation of Wnt signaling pathway, the expression of the target gene MMP-2 and MMP-9 in OA mice increased (Figure 2A, 2E and 2F).

The β-catenin, the core protein in the Wnt signaling pathway, was transferred from the cytoplasm to the nucleus after activation. The results showed that in the OA model, when compared to the normal group, the β-catenin in the nucleus increased (Figure 2B) and the intensity of Klotho immunostaining in the articular cartilage of the OA mice decreased (Figure 2I).

### In cartilage tissue of OA model, the binding of Dvl to Frizzled, Klotho to Wnt1, Wnt4, Wnt 7a and β-catenin to MMP2, 9 promoters were detected

**Figure 3:**
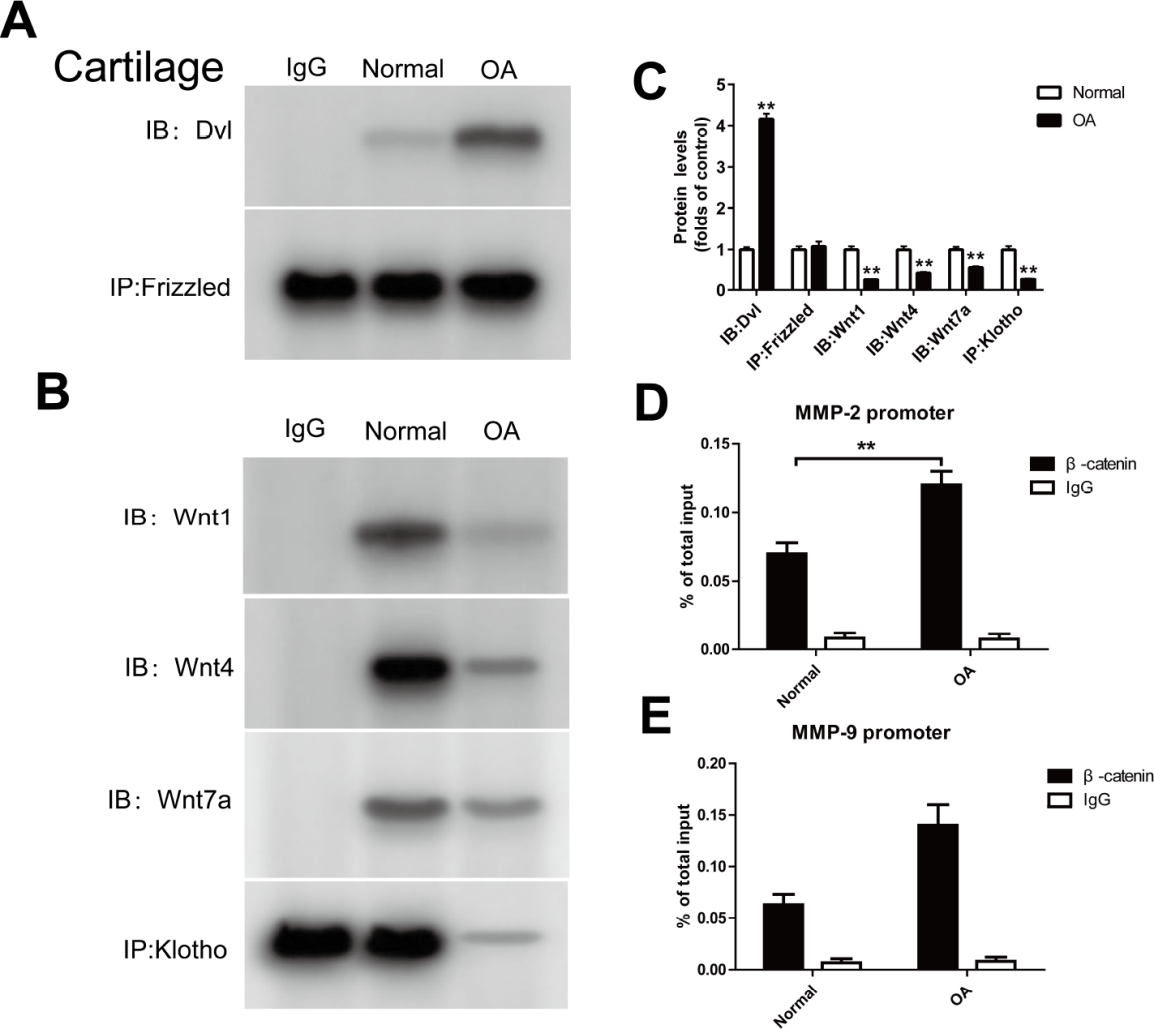
**Klotho binds to Wnt and blocks Wnt-mediated gene transcription.** In cartilage tissue, the binding of Dvl to Frizzled, Klotho to Wnt1, Wnt4, Wnt7a and β-catenin to MMP2, 9 promoters were detected. (**A**, **B**) The Co-IP method was used to detect the binding of Dvl to Frizzled, and Klotho to Wnt1, Wnt4, Wnt7a in cartilage tissue after operation, (**C**) and quantitative data for Dvl to Frizzled, and Klotho to Wnt1, Wnt4, Wnt7a are presented. (**D**, **E**) The binding of β-catenin to MMP2, 9 promoters was detected by ChIP method. Data are the mean ±SD, n=8 mice.. *P < 0.05, **P < 0.01.

Dishevelled (Dvl), a key protein in the Wnt signaling pathway, can transfer the Wnt signal from the receptor to the downstream effector. In the classical Wnt signaling pathway, Dvl is convoked to the cell membrane by the receptor Fizzled, and wherefore binds to the axin/GSK3/APC complex, which consequently inhibits the degradation of β-catenin in the cytoplasm. In the cartilage tissue of OA mouse model, the Dvl and Fizzled binding markedly increased (Figure 3A) by Co-IP method.

Previous experiments suggest that Klotho can bind with Wnt1, 4 and 7a to form a complex, inhibits the activation of Frizzled, LRP 5/6 complex by Wnt, and therefore plays a similar role to Wnt1,4,7a antagonist. In the OA model, we also found that Klotho and Wnt1, 4 and 7a binding decreased when compared to normal cartilage tissue (Figure 3B).

ChIP method was used to detect the binding of β-catenin to MMP 2, 9 promoters. The results showed that the combination of TCF and MMP 2,9 promoters increased in the OA model (Figure 3D, 3E).

### Expression of the Klotho, wnt/β-catenin signal pathway and its target gene in the chondrocytes stimulated by different CTS in the vivo experiment

**Figure 4:**
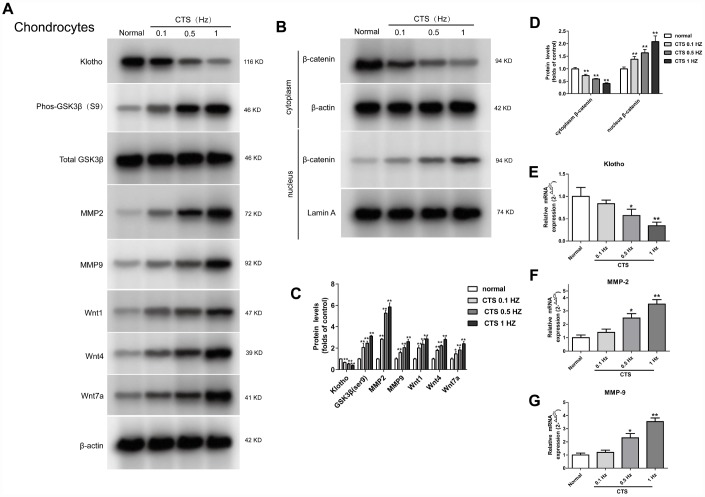
**Up-regulation of Wnt signaling and down-regulation of Klotho in chondrocytes treated by CTS.** Chondrocytes were were pretreated with cyclic tensile strain (0.1-1Hz) for 48 hours Expression of Klotho, Wnt1, Wnt4 and Wnt7a in the cell supernatant and GSK3β, p-GSK3β, MMP2, MMP9 and β-catenin (cytoplasm/nucleus) in the cell of mouse cartilage. (**A**) Western blot analyses show that CTS induced these proteins expression in a tension-dependent manner. (**B**) Cytocol/nuclear separation was undertaken to assess the enrichment of β-catenin in the nuclei in chondrocytes treated by CTS. The upregulation level of β-catenin localized in nucleus was more obvious than that remained in cytosol in CTS groups than control group. Representative Western blots (**A** and **B**) and quantitative data (**C** and **D**) are presented. *P<0.05 versus controls (n=3). (**E**) The RT-PCR results showed periodic CTS dependent inhibition of Klotho expression. (**F**, **G**) The results of RT-PCR showed that the expression of MMP-2, MMP-9 was stimulated by periodic CTS dependent stimulation. Results were obtained via the expression of three individual experiments performed in triplicate for each condition. *P < 0.05, **P < 0.01.

The same findings were observed in vivo experiment. To further examine the expression of Klotho in chondrocytes and simulate the cartilage stimulated by stress in vivo, we established a model of OA chondrocytes exposed to CTS *in vitro*. Klotho protein level in the supernatant of chondrocytes was decreased with the increase of CTS intensity (Figure 4A and 4E) after 48h treatment with CTS (0.1, 0.5 and 1HZ). Western blot results showed that expression of Phos-GSK3β (s9) and Wnt1, Wnt4 and Wnt 7a in the supernatant of Wnt pathway was increased with the increase of CTS intensity (Figure 4A), and the nuclear Wnt pathway core protein β-catenin was decreased (Figure 4B), while the expression of Wnt target gene MMP-2 and MMP-9 increased (Figure 4F, 4G).

### *In vitro*, CTS stimulated chondrocytes were used to detect the binding of Dvl to Frizzled, klotho to Wnt1, Wnt4 and Wnt 7a in the cell supernatant and cell, and the effect of β-catenin on the activity of MMP2, 9 promoters were detected in the vitro CTS stimulated chondrocytes

**Figure 5:**
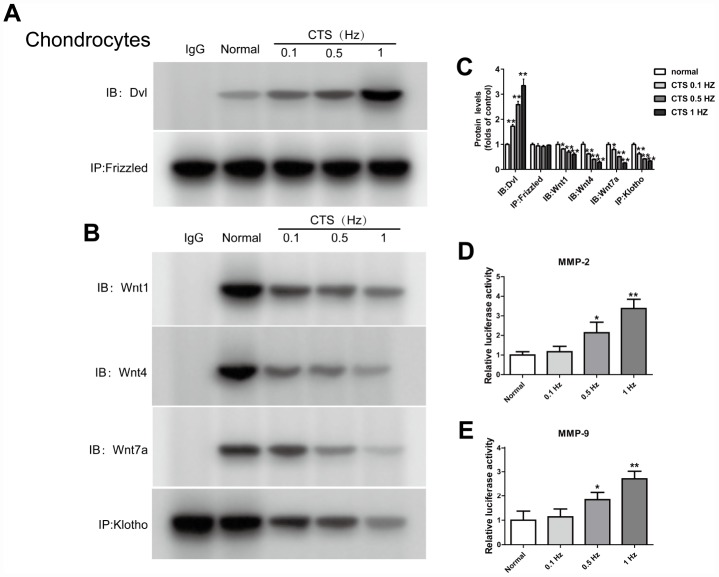
**CTS influenced Wnt/β-catenin signaling pathway, Klotho binds to Wnt and blocks Wnt-mediated gene transcription.** Chondrocytes were incubated with or without CTS(0.1, 0.5.1 HZ) for 48 hours. the binding of Dvl to Frizzled was induced by CTS in a tension-dependent manner, Klotho to Wnt1, Wnt4, Wnt7a was adversed. (**A**, **B**) Detection of Dvl and Frizzled binding and (**B**) Klotho and Wnt1, 4, 7a binding in supernatant by Co-IP, quantitative data (**C**) is presented. *P< 0.05 versus controls (n=3). (**D**, **E**) The effect of β-catenin on the activity of MMP-2 promoters and MMP-9 promoters was in a tension dependent manner. Results were obtained via the expression of three individual experiments performed in triplicate for each condition. *P < 0.05, **P < 0.01.

*In vitro* experiments, the binding level of Dvl and Frizzled in the chondrocytes increased with the increase of the CTS intensity (Figure 5A). In contrast, the binding level of Klotho and Wnt 1, Wnt 4 and Wnt 7a in the supernatant of chondrocytes was decreased with the increase of CTS intensity (Figure 5B). And the effect of β-catenin on the activity of MMP2, 9 promoters was increased with the increase of CTS intensity (Figure 5D, 5E).

### Expression of wnt/β-catenin signaling pathway and its target gene in chondrocytes over-expression of Klotho

**Figure 6:**
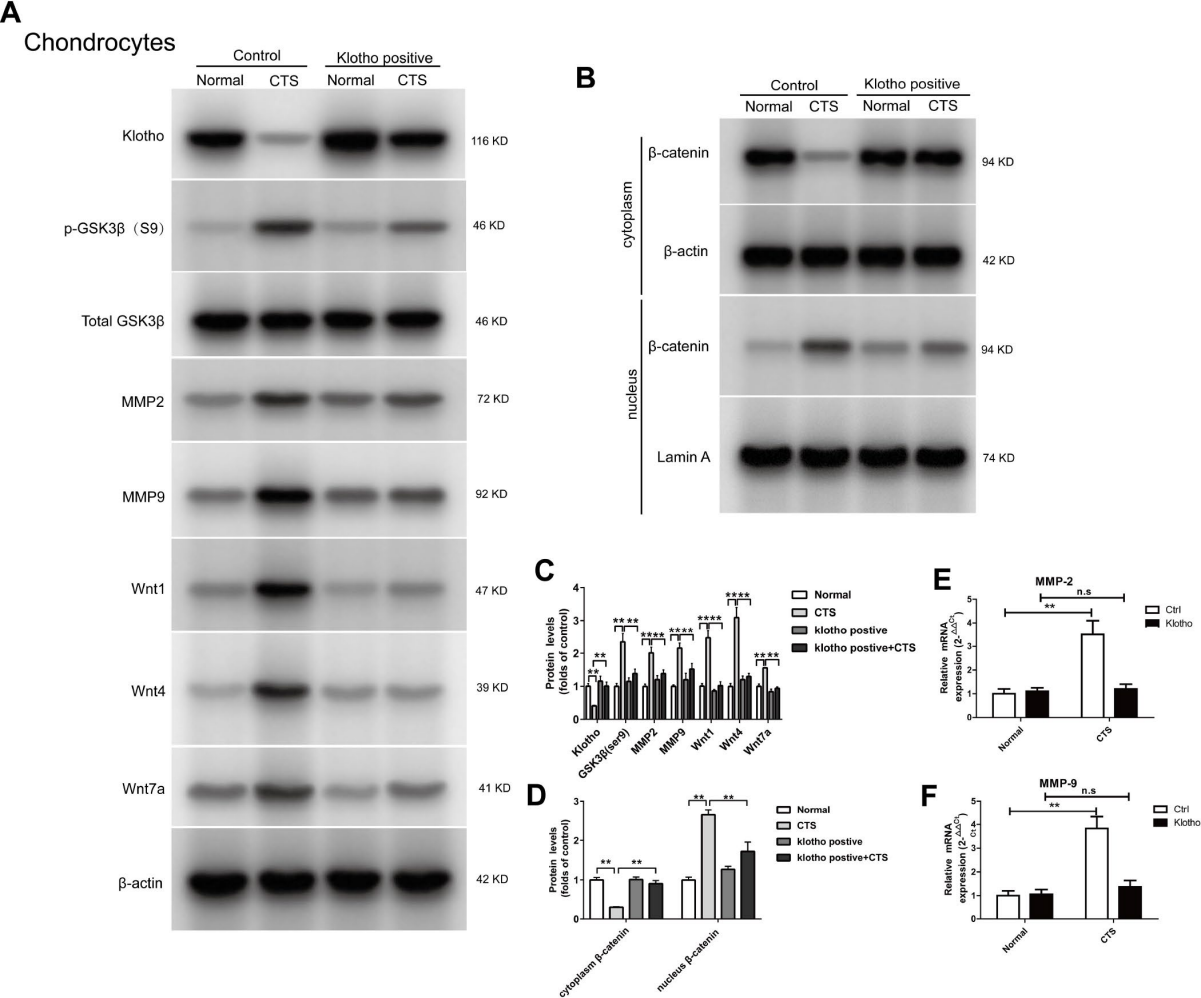
**Klotho inhibits Wnt-mediated β-catenin activation and represses its target genes in vitro.** Chondrocytes were transfected with empty vector or Klotho expression vector as indicated, followed by incubation with or without CTS(1 HZ) for 48 hours. Western blot (**A**, **B**) and quantitative data (**C**, **D**) for Wnt1, Wnt4 and Wnt7a in the cell supernatant and GSK3β, p-GSK3β, MMP2, MMP9 and active β-catenin are presented. Klotho abolished CTS-mediated Wnt actions. (**E**, **F**) RT-PCR was used to detect the expression of MMP-2 and MMP-9 mRNA in different groups. Results were obtained via the expression of three individual experiments performed in triplicate for each condition. *P < 0.05, **P < 0.01.

The regulation of Klotho over-expression on the formation of OA induced by CTS was further studied. First, the chondrocyte was transfected with Klotho-overexpressing lentiviral vector or negative control lentivirus vector. The results showed that in the chondrocytes over-expression of Klotho, after increase of the Klotho expression, CTS could not increase the expression of Wnt signal pathway related proteins, including Phos-GSK3β (S9) in cells, and Wnt1, Wnt4 and Wnt7a) in the supernatant (Figure 6A). Over-expression of Klotho inhibited CTS's promotion of β-catenin from the cytoplasm to the nucleus (Figure 6B). The over-expression of Klotho reduced the transcription level of downstream target gene MMP-2 and MMP-9 (Figure 6A, 6E, 6F).

### In chondrocytes overexpressed with klotho, the binding of Klotho to Wnt1, Wnt4 and Wnt7a, the binding of Dvl to Frizzled, and the effect of β-catenin on the activity of MMP2 and MMP9 promoters were observed

**Figure 7:**
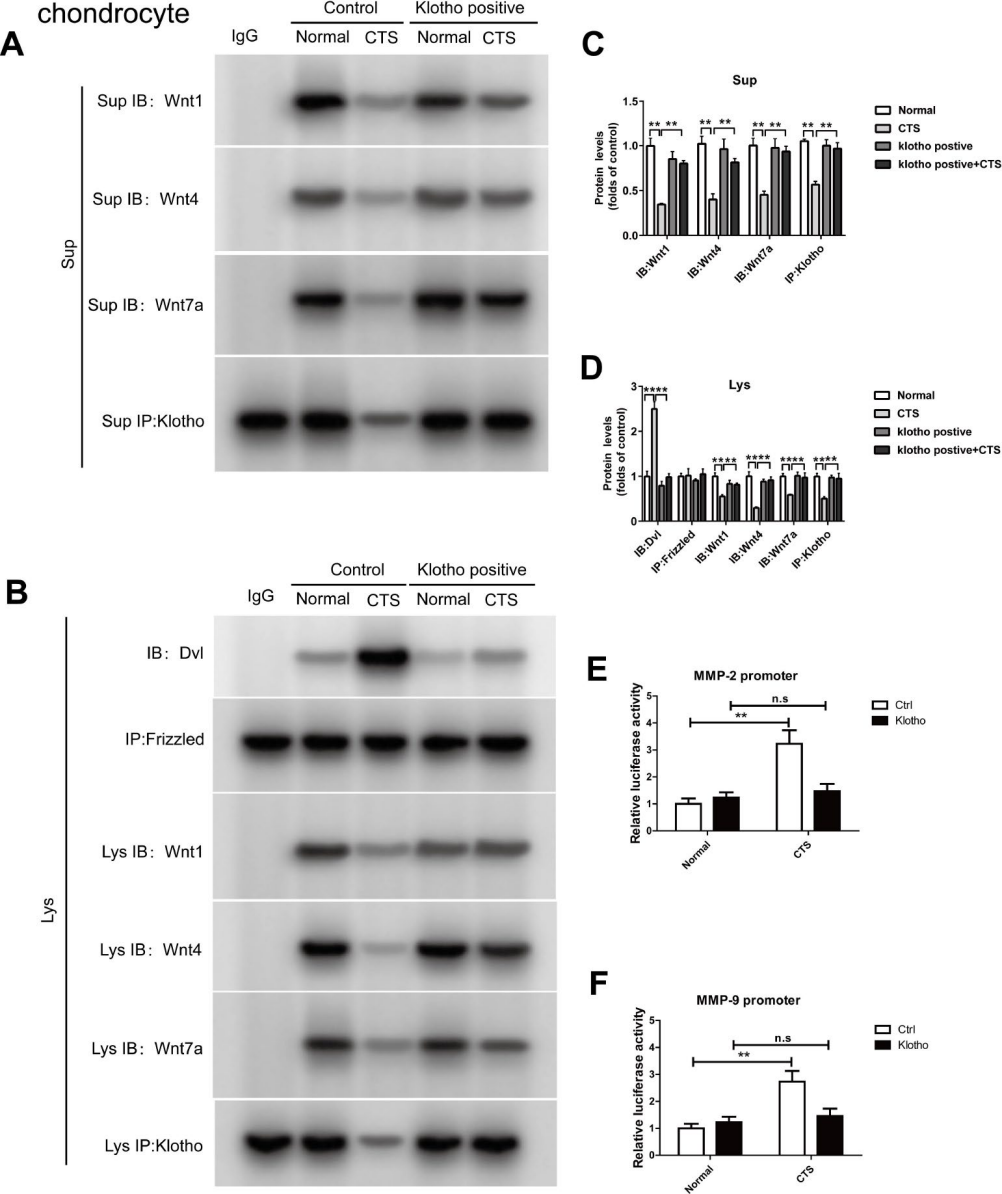
**Over-expressed Klotho binds to Wnt and blocks Wnt-mediated gene transcription in vitro.** Chondrocytes were incubated with or without CTS(1 HZ) for 48 hours. Overexpression of klotho attenuated the activity of the wnt signaling pathway (**A**) Co-IP method was used to detect the binding of Klotho and Wnt1, Wnt4 and Wnt7a in different groups of cells supernatant. (**B**) Co-IP method was used to detect the binding of Klotho and Wnt1, Wnt4, Wnt7a, the binding of Dvl to Frizzled in different groups of cells, quantitative data (**C**, **D**) is presented. *P,0.05 versus controls (n=3), The cell lysates were immunoprecipitated by β-catenin antibody. qPCR analyses the enrichment of MMP-2, MMP-9-specific DNA in chondrocytes. (**E**, **F**) Overexpression of klotho blocks the binding of β-catenin on MMP-2 and MMP-9 promoter activity. Results were obtained via the expression of three individual experiments performed in triplicate for each condition. *P < 0.05, **P < 0.01.

In the chondrocytes overexpressed with Klotho, Co-IP experiments confirmed that Klotho reversed the combination of Dvl and Frizzled induced by CTS (Figure 7A). On the other hand, under the action of CTS, the binding of Wnt1, Wnt4, Wnt7a to Klotho in chondrocytes with over-expression of Klotho was increased (Figure 7A). The cell supernatant showed the same result (Figure 7B). Over-expression of Klotho reduced the binding level of downstream target gene β-catenin and MMP-2 and MMP-9 promoters by luciferase assay (Figure 7E, 7F).

### Expression of Klotho, Wnt1, Wnt4 and Wnt7a in the supernatant, GSK3β, p-GSK3β, MMP2, MMP9, MMP-7, active β-catenin and β-catenin (cytoplasm/nucleus) in β-catenin silencing chondrocytes

**Figure 8:**
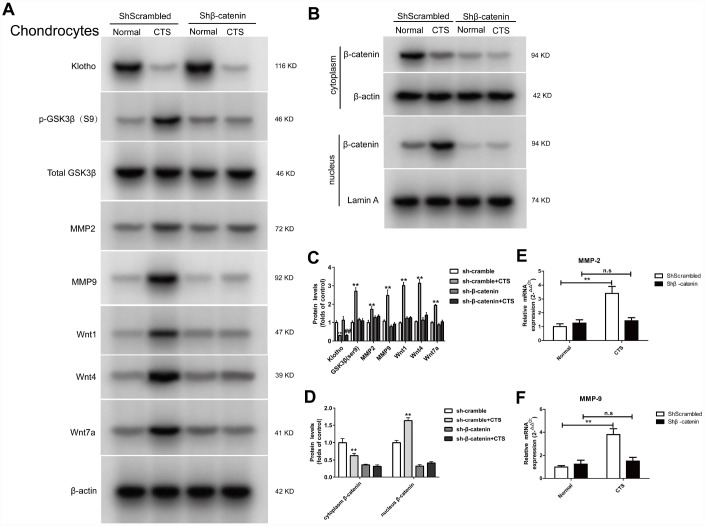
**Expression of Klotho, Wnt1, Wnt4, Wnt7a in the cell supernatant and GSK3β, p-GSK3β, MMP2, MMP9, MMP-7, β-catenin (cytoplasm/nucleus) in the cell of chondrocytes knockdown of β-catenin.** (**A**) The protein expression detection of Klotho, Wnt1, Wnt4, Wnt7a in the cell supernatant and Phos-GSK3β (S9), GSK3β, MMP2, MMP9, and (**B**) β-catenin (cytoplasm/nucleus) by Western Bolt, quantitative data (**C**) is presented. *P,0.05 versus controls (n=3), (**E**, **F**) RT-PCR was used to detect the expression of MMP-2 and MMP-9 mRNA in different groups. Results were obtained via the expression of three individual experiments performed in triplicate for each condition. *P < 0.05, **P < 0.01.

β-catenin is a multifunctional protein in the cytoplasm and a key molecule regulating gene transcription in Wnt signaling pathway. First, the chondrocyte was transfected with β-catenin low expressing lentiviral vector or negative control lentivirus vector. The results showed that there was no markable change in the expression of Klotho in the supernatant of chondrocytes between the β-catenin silencing chondrocytes and the normal cells, both decreased (Figure 8A). However, the expression of Wnt signal-related proteins p-GSK3β, WNT1, WNT4 and WNT7a decreased after silencing β-catenin, indicating that the Wnt signaling pathway was silent after β-catenin silencing. After this silencing, the β-catenin in chondrocytes could not be transferred from cytoplasm to the nucleus in the presence of CTS (Figure 8B). The silencing β-catenin has reduced the transcription level of downstream target gene MMP-2, MMP-9 (Figure 8A, 8E, 8F). These results suggest that. Klotho is in the upstream of WNT/β-catenin signaling pathway, and Klotho regulates OA through Wnt signaling pathway

### In the β-catenin silencing chondrocytes, the binding of Klotho to Wnt1, Wnt4, Wnt7a, the binding of Dvl to Frizzled, and the effect of β-catenin on the activity of MMP2 and MMP9 promoters were observed

**Figure 9:**
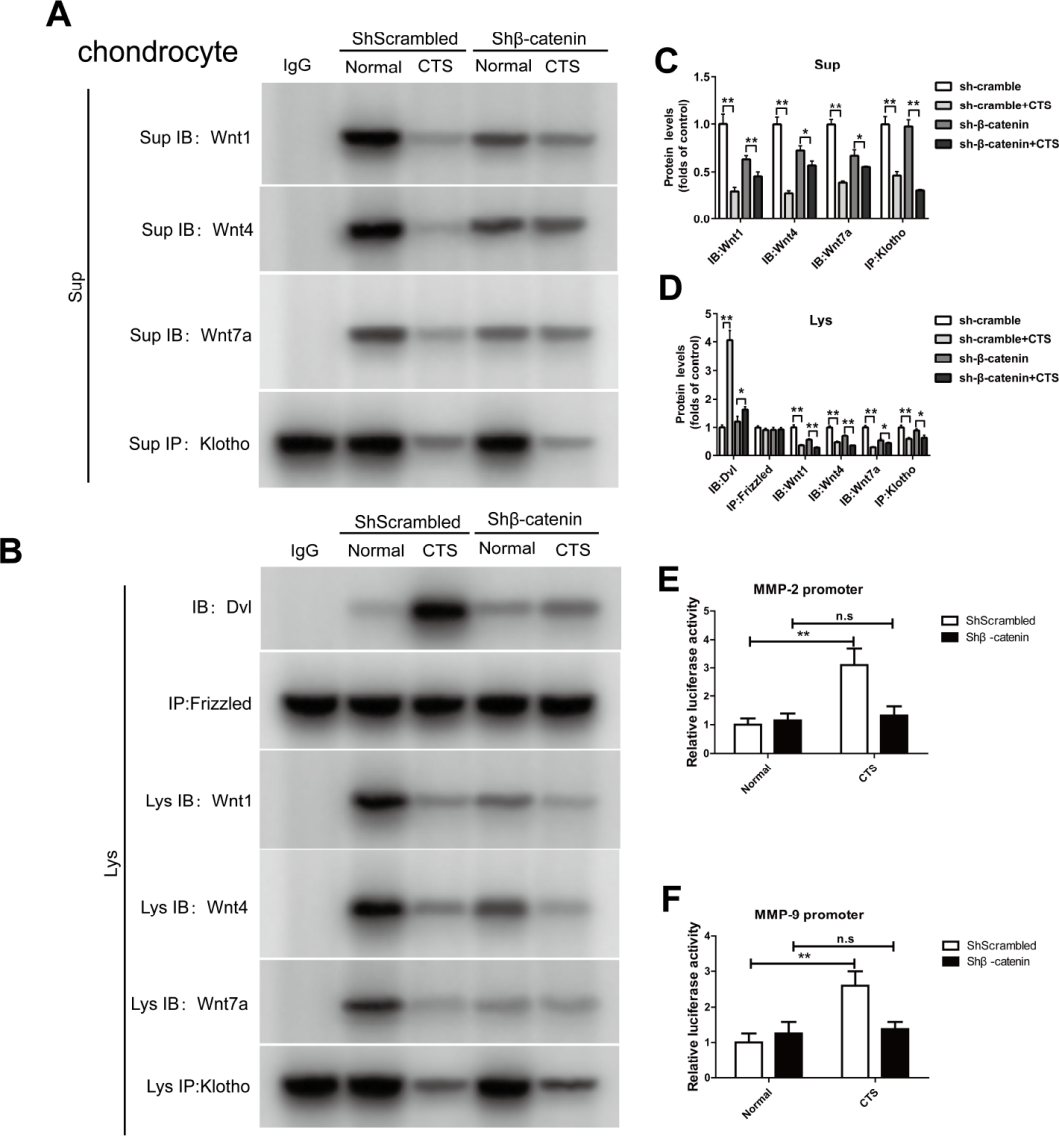
**In chondrocytes knockdown of β-catenin, the binding of Dvl to Frizzled, Klotho to Wnt1, Wnt4, Wnt7a and β-catenin to MMP2, 9 promoters were detected.** (**A**) Co-IP method was used to detect the binding of Klotho and Wnt1, Wnt4, Wnt7a in different groups of cells supernatant. (**B**) Co-IP method was used to detect the binding of Klotho and Wnt1, Wnt4 and Wnt7a, the binding of Dvl to Frizzled in different groups of cells. quantitative data (**C**, **D**) is presented. *P,0.05 versus controls (n=3), The cell lysates were immunoprecipitated by β-catenin antibody. qPCR analyses the enrichment of MMP-2, MMP-9-specific DNA in chondrocytes. (**E**, **F**) MMP-2 and MMP-9 promoter luciferase activity decreased after silencing β-catenin. Results were obtained via the expression of three individual experiments performed in triplicate for each condition. *P < 0.05, **P < 0.01.

In the β-catenin silencing chondrocytes, Co-IP assay confirmed that there was no markable change in the binding of Dvl to Frizzled with or without CTS stimulation (Figure 9A). Silencing β-catenin led to the decrease of Wnt1, 4 and 7a expression, and the binding of Wnt1, Wnt4 and Wnt7a to Klotho in β-catenin silencing chondrocytes was also reduced by CTS, which was same in the cell supernatant (Figure 9A, 9B). Silencing β-catenin reduced the binding level of downstream target gene β-catenin, MMP-2 and MMP-9 promoters by luciferase assay (Figure 9E, 9F).

## DISCUSSION

OA is a common multivariate joint disease, in which caducity and overloading are main risk factors. Although the pathophysiology of OA involves cartilage, bone and synovial tissue, the primary feature of OA is the progressive degeneration of articular cartilage. Studies suggest that the progressive joint destruction in OA is caused by over-activation of Wnt signal transduction [12–17]. The Wnt signaling pathway is an effective regulator of bone and cartilage homeostasis and also plays an important role in human joint diseases [23, 24].

In many diseases, the down-regulation of Klotho increases the inflammation and oxidative stress [25–27]. OA is known to be a degenerative joint disease closely related to caducity. However, there is little research into the participation of Klotho in the pathogenesis of OA. This study suggests that in both vitro and vivo OA model, the expression of Klotho decreases (Figures 2, 4). Similarly, previous studies show that the secreted α-Klotho can maintain the balance of cartilage tissue by inhibiting the catabolism axis of NOS2 and ZIP8-MMP13. Additionally, the expression of Klotho in chondrocytes stimulated by IL-1β also decreases in OA mice [7].

Previous studies have shown that Klotho expressed in renal tubular epithelial cells can bind to Wnt1, 4 and 7a to form a complex, which inhibits the activation of Frizzled, LRP5/6 complex by Wnt, and wherefore acts as a Wnt1, 4 and 7a antagonist in renal injury model [11]. Our results confirm that *in vitro* and *vitro* experiments of OA cartilage tissue, Klotho can also be combined with Wnt1, 4, 7a (Figures 3 and 5). At the same time, in OA model, the expression of Wnt1, 4 and 7a increases after the decrease of Klotho expression, consequently leading to the activation of Wnt signaling pathway (Figures 2 and 4).

The promoter of Wnt signal transduction pathway is the Wnt family protein, which is a secretory glycoprotein; besides, 19 family members have been found all containing 24 conserved cysteine residues [28, 29]. Among the Wnt family proteins, Wnt1, Wnt3a, Wnt4, Wnt7a and Wnt7b have been proved to be involved in the classical pathway of β-catenin upstream gene activation [30–32]. The classical Wnt pathway describes a series of reactions when the Wnt protein binds to the Frizzled receptor family on the cell surface, including the activation of dishevelled receptor family proteins and the changes of β-catenin levels in the nucleus. Dishevelled (DSH) is a key component in the membrane associated Wnt receptor complex, which is activated by binding to Wnt and wherefore inhibits downstream protein complexes, including Axin, GSK-3, and APC proteins. The Axin/GSK-3/APC complex can promote the degradation of intracellular signal molecule β-catenin. If this β-catenin degradation is inhibited, the β-catenin could exist stably in the cytoplasm, and some β-catenin could enter into the nucleus to interact with the TCF/LEF transcription factor family and thus promote the expression of specific genes [33, 34].

We observed the four core processes in the activation of WNT/β-catenin signaling pathway in the OA arthritis model, including the phosphorylation of GSK3β, the binding of Dvl to Frizzled protein, β-catenin enriched and transferred to nucleus, as well as the binding of β-catenin, TCF/LEF transcription factor and MMP promoters in nucleus.

GSK3β is a Serine/Threonine protein kinase, which, in the absence of Wnt signal, is able to add the phosphate group to the Serine/Threonine residues at the N-terminus of the β-catenin, wherefore the phosphorylated β-Catenin is covalently modified by the β-TRCP ubiquitination and then degraded by the proteasome. After activation of the Wnt signal, the activity of GSK3β decreases after the phosphorylation, which weakens its ability to degrade β-catenin. Our results showed that the level of p-GSK3β increased in OA model in vivo and vitro (Figures 2A, 4A). The results additionally showed that, in this model, with the decrease of Klotho, Dvl, a positive regulator of Wnt signal transduction located in the downstream of Frizzled receptor and upstream of β-catenin, increased its binding to Frizzled; whilst *in vitro* experiment, this binding was CTS intensity dependent.

Some types of matrix metalloproteinase (MMPs), such as MMP-2 and MMP-9), are direct transcriptional targets of Wnt/β-catenin signaling pathway [15, 18]. MMP-2 and MMP-9 are both important members of the MMPs family and important enzymes in the degradation of articular cartilage matrix. The MMP-9 plays a major role in destroying cartilage matrix, exposing collagen and destroying the reticular structure of collagen. The MMP- 2 secreted into the extracellular matrix in the form of proenzyme, which can degrade the important components of articular cartilage in the extracellular matrix, such as type I, IV, V and type X collagen, laminin, fibronectin and proteoglycan [19–21]. The results found that in vivo and vitro model of OA, with the decrease of Klotho, the expression of MMP-2 and MMP-9 increased; whilst *in vitro* experiment, this increase was CTS intensity dependent.

In further study, the binding of Wnt1, 4 and 7a to Klotho in chondrocytes increased significantly in cell supernatant and cells after chondrocyte over-expression of Klotho by using Klotho over-expression lentivirus vector. Under the action of CTS, Klotho reversed the activation of WNT/β-catenin signaling pathway (Figures 6, 7), indicating that the Klotho regulates OA through WNT/β-catenin. Similarly, Soluble Klotho (50, 100, 200ug/ml) inhibits Wnt-mediated β-catenin activation and represses its target genes in vitro in a dose-dependent manner (Supplementary Figure 1).

As an important component in Wnt signaling pathway, the activation of β-catenin in the articular chondrocytes participates in the transition from normal articular chondrocytes to terminal differentiation chondrocytes during OA development. Expression of β-catenin is up-regulated in OA cartilage, and the over-expression of β-catenin in the articular chondrocytes makes adult mice susceptible to OA phenotype [14, 15, 35]. In addition, β-catenin can promote bone formation and participate in abnormal differentiation of osteoblasts and ectopic formation of cartilage. If β-catenin is inactivated, the mesenchymal cells would differentiate into chondrocytes rather than osteoblasts, indicating that β-catenin can regulate both intramembranous and endochondral osteogenesis [36]. When the β-catenin is transferred to the nucleus and bound to TCF/LEF, it activates the downstream target genes, such as C-myc, MMP-3, MMP-13, MMP-7, MMP-9, and bone morphogenetic protein 2 genes for transcription. All these genes are in a state of abnormal activation in OA, and participate in the occurrence and development of OA [15]. In the model of OA *in vivo* and *in vitro*, the content of β-catenin in the nucleus increased and over-expression of Klotho could reduce the β-catenin transfer into the nucleus. Further study showed that after the β-catenin in chondrocytes was silenced by low expression of lentivirus vector, the reduced Klotho could not increase MMP expression through Wnt signaling pathway under the action of CTS (Figures 8, 9).

In conclusion, this study suggests that Klotho deficiency is a common pathological feature in OA and the biological effect of this deficiency is related to the activation of WNT/β-catenin signaling pathway. Klotho can combine with Wnt and functions as an effective and endogenous Wnt antagonist. Klotho deficiency will inevitably result in articular cartilage injury through Wnt /β-catenin signal transduction. Wherefore, we believe that Klotho may be a potential target for the treatment of OA cartilage disease.

## MATERIALS AND METHODS

### Reagents

Monoclonal antibodies against Phos-GSK3β (S9), GSK3β, MMP2, MMP9, Wnt1, Wnt4, Wnt7, Frizzled and β-catenin were provided by Abcam (Cambridge, UK). Anti-Dvl Antibody was provided by Cell Signaling Technology (Danvers, MA and USA). Antibodies against Klotho was provided by Abcam and Santa Cruz Biotechnology Fetal bovine serum and DMEM low- and high-glucose medium were procured form HyClone (Logan, UT and USA) Phosphate-buffered saline (PBS), cytoplasmic and membrane protein extraction kit, total protein extraction kit, RIPA buffer and PMSF were procured from Beyotime Biotechnology (Nantong, China). ChIP Assay Kit was obtained from Beyotime Biotechnology, (Shanghai, China). Pierce™ Classic Magnetic IP/Co-IP kit was obtained from Thermo Fisher (Shanghai, China). Acin blue staining kit was provided by Nanjing Jiancheng Bioengineering Institute (Nanjing, China).

### Animals

Twenty-four C57/6J mice (19-21gm, 3-month-old) were obtained from the Animal Inc., affiliated to Nanjing Medical University (Nanjing, China). These mice were separated into two groups randomly (control group and OA group respectively), consisting of 12 mice for each, which were kept in four animal cages (six mice per cage) in a temperature-controlled room (21–23°C) with free access to water and food. All these animal procedures were approved by the Animal Research Ethics Committee of Nanjing Medical University.

### Anterior cruciate ligament transaction (ACLT)

ACLT surgery was used to induce OA in adult male C57/6J mice. Briefly, all mice were anesthetized with chloral hydrate. A medial parapatellar approach was adopted to expose the right knee joint. After that, a transection was conducted with micro-scissors in the anterior cruciate ligament (ACL) in the OA group with a positive anterior drawer sign to confirm the completeness of transection. In the control group, the arthrotomy was conducted while the transection of ACL was not conducted. After surgery, the mice were released from the cages for 30 minutes on a daily basis.

### HE and alcian blue staining

The mice were sacrificed by cervical dislocation 12 weeks after surgery and under anesthesia. The knee joint cavity was exposed by separating the patella. The fixed specimens were decalcified for 3 weeks by using 10% ethylenediaminetetraacetic. Upon decalcification, the samples were embedded in paraffin; then the standard serial sections of 3μm each were cut in sagittal plane before the sections were stained by hematoxylin and eosin (H&E), and Alcian blue staining.

### Immunohistochemistry

For immunohistochemical analysis of Klotho and β-catenin expression in articular cartilage of mice, the paraffin-embedded tissues in full-thickness were processed in this study. A blocking serum (Vectastain ABC Kit, Vector Laboratories, Inc., Burlingame, CA, USA) was used to incubate the slides for 60 minutes. Then the slides were overlaid with primary antibody against Klotho and β-catenin for 2 hours at room temperatures after being blotted. Subsequently, the sections were added with biotinylated secondary antibodies before they were processed by the streptavidin-biotin staining technique (DAB Kit, Invitrogen, Paisley, UK), which is peroxidase-labeled.

### Chondrocyte culture, cyclic tenisle strain

The first step is to separate chondrocytes from articular cartilage of mice’s knee joints. In addition, the articular cartilage tissues were digested for 30 minutes by using 0.25% trypsin after being cut into small pieces (<1 mm). After this step, digestion using 0.2% Type II collagenase was performed for 3 hours. DMEM/F12 media, antibiotics and 10% fetal bovine serum (FBS) were then used to culture the released cells. After the confluence increased up to 80%, the cells were subjected to cyclic tensile strain for 48 hours at 20% elongation with a 0.1-1 Hz sinusoidal curve indicated by a Flexcell1 FX-5000 Tension System following the user’s instructions (Flexcell International Corporation, Burlington, NC, USA). In order to preserve the chondrocyte phenotype, only the cells not exceeding two passages were adopted.

### Cell transfection

Both empty lentiviral vectors and lentiviral vectors encoding Klotho or β-catenin were acquired from Genechem (Shanghai, China). Lentiviral transfection was conducted by following the manufacturer’s instructions. Green fluorescent protein (GFP) was used to assess the infection efficiency by using flow cytometry. 5μg/ml puromycin (Sigma-Aldrich, USA) was used to select stably transfected cells after 48 hours.

### Western blotting analysis

Total protein from cell supernatant, chondrocyte and cartilage was prepared by RIPA buffer, the nuclear protein was isolated by using Nuclear Protein Extraction Kit (Beyotime) strictly in accordance with the Kit instructions, the protein mixtures were separated by gel electrophoresis in sodium dodecyl sulfate-polyacrylamide, which were then translated to nitrocellulose membranes. Briefly, 10% SDS-PAGE was used to extract 15μg plasma proteins from the cartilage tissue and chondrocytes. In addition to that, the proteins were electroblotted onto a polyvinylidene difluoride membrane with a thickness of 0.45 mm (Millipore, Bedford, MA, USA), which were then blocked via 5% non-fat dry milk in Tris-buffered saline with Tween 20 for one hour. Incubation of these membranes was then performed at 4 °C overnight with Klotho, Phos-GSK3β (S9), GSK3β, MMP2, MMP9, Wnt1, Wnt4, Wnt7 and β-catenin antibodies at room temperatures for one hour. To visualize antigen-antibody complexes, the enhanced chemiluminescence assay (Thermo Scientific, Pierce, Rockford, IL, USA) was applied.

### Co-immunoprecipitation

Immunoprecipitation lysis buffer (30mm Tris-HCl, 150mm NaCl, 1% Triton X-1002 mm EDTA, 10% glycerol and 1×protease inhibitor) was used to homogenize cartilage tissue on ice before a short step of brief pulses of hand sonication. Following the step of 30-minute incubation on ice, a centrifugation operation was conducted for the lysate and then Protein A/G PLUS-agarose beads were used to preclear the supernatant by incubation at 4 °C. After that, 2μg antibody was used to incubate the precleared supernatant on a rocker at 4 °C for 4 hours against Klitho. The centrifugation was then performed to collect immune-precipitates at 1,000×g for five minutes after adding beads for one hour additionally. Immunoprecipitation lysis buffer was then used to wash the obtained immune-precipitates for 3 times, which were subsequently centrifugated after each wash. 2×sample buffer was used to resuspend and boil the pellet before Western blot was used in the analysis of Wnt1, 4 and 7a by the method as described above.

### Quantification of mRNA and qRT-PCR

TRIzol reagent (Invitrogen) was used to extract entire RNA from chondrocytes, which was subsequently reversely transcribed into cDNA with the tool of PrimeScript RT reagent kit (TaKaRa, Dalian, China). SYBR Premix Ex Taq II reagents which were provided by TaKaRa (Dalian, China) were used to analyze PCR in a quantitative real-time manner, following the manufacturer’s instructions. 20μL mixture with 2μL cDNA was used to demonstrate PCR results. Specific primers (TaKaRa) were used to amplify Klotho cDNAs. Glyceraldehyde-3-phosphatedehydrogenase (GADPH) was used to normalize gene expression, which was then derived by the 2-DDCT approach. These are the primer sequences: Klotho: forward, 5′-ACTACGTTCAAGTGGACACTACT-3′ and reverse 5′-GATGGCAGAGAAATCAACACAGT-3′; MMP-2: forward, 5′-CAAGTTCCCCGGCGATGTC-3′ and reverse 5′-TTCTGGTCAAGGTCACCTGTC-3′; MMP-9: forward, 5′-CTGGACAGCCAGACACTAAAG-3′ and reverse 5′-CTCGCGGCAAGTCTTCAGAG-3′.

### Chromatin immunoprecipitation (ChIP) assay

ChIP assays were performed essentially as described [22]. Nuclear protein was then isolated from cartilage tissue and chondrocyte. Diluted soluble chromatin fractions were immunoprecipitated with 1μg polyclonal anti-β-catenin antibody or control mice immunoglobulin G (IgG). The histone-DNA crosslinks were reversed by 4-hour incubation at 65°C. PCR was used to analyze the DNA fragments from ChIP assays. These are the primer sequences: MMP-2: forward, 5′-GAGGTCGCTTTCTTTGCCATCT-3′ and reverse 5′-AGCGACTCCATCTTGAACAGG-3′; MMP-9: forward, 5′-CACTGTATCCTTGACCTTCTTTCTGG-3′ and reverse 5′-GCTTCCTCTCCCTGCTTCATCTG-3′.

### Luciferase assay

Cells were co-transfected by using Lipofectamine 2000 reagent (Life Technologies) with mmp2/mmp9-Luc luciferase reporter constructs (1.0μg) plus shScrambled or shβ-catenin expression plasmid (2.0 μg) (Genechem), respectively. A fixed amount (0.1 μg) of internal control reporter Renilla reniformis luciferase driven under a thymidine kinase promoter(pRL-TK;Promega) was also cotransfected for normalizing the transfection efficiency. Luciferase assay was carried out using a dual luciferase assay system kit according to the manufacturer's protocols (Promega). Relative luciferase activity was reported as fold induction over controls after normalizing for transfection efficiency.

### Statistics

Statistics have shown ±SEM of the triplicate values for each experiment. One-way analysis of variance (ANOVA) was used to assess the difference between experiment and control groups. When the P value is <0.05, the difference is significant.

## Supplementary Material

Supplementary Figure 1
