# Supplementary material for: Loss of Klotho contributes to cartilage damage by derepression of canonical Wnt/β-catenin signaling in osteoarthritis mice
Source: Aging (Albany NY). 2019 Dec 30;11(24):12793–809. doi: 10.18632/aging.102603 (PMC6949099; doi:10.18632/aging.102603)
Supplement: Supplementary Figure 1 [file aging-11-102603-s001..pdf]

SUPPLEMENTARY FIGURE

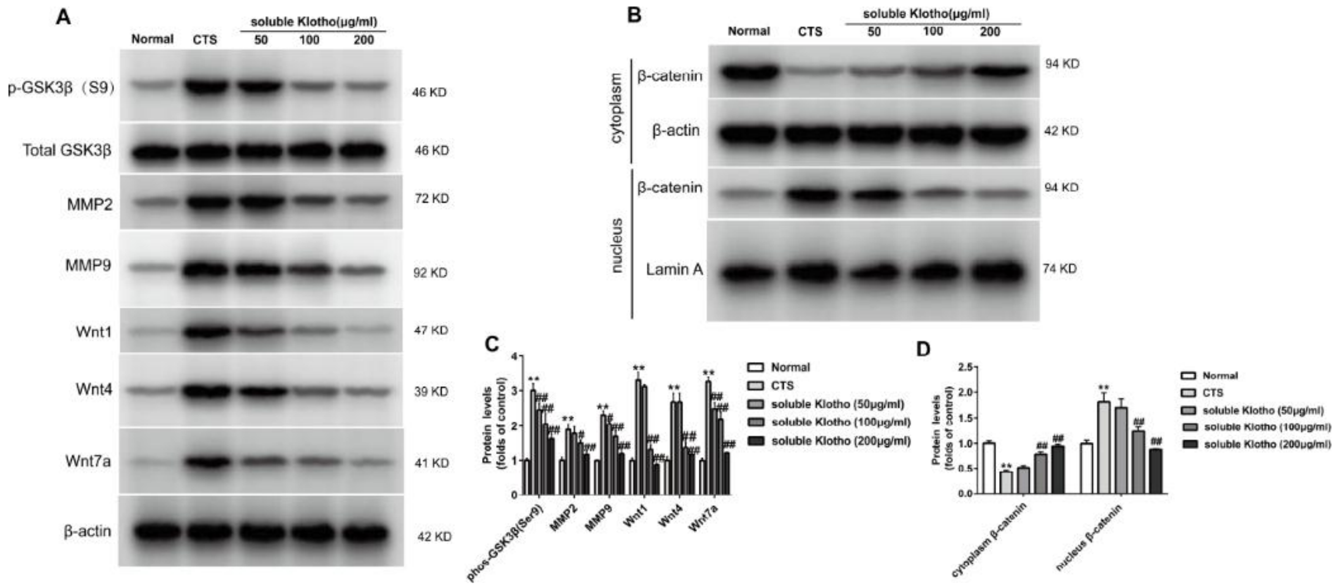

Supplementary Figure 1. Soluble Klotho inhibits Wnt-mediated β-catenin activation and represses its target genes in a dose-dependent manner. Expression of Wnt1, Wnt4 and Wnt7a in the cell supernatant and GSK3β, p-GSK3β, MMP2, MMP9 and β-catenin (cytoplasm/nucleus) in the cell of chondrocytes treatde with soluble Klotho (50, 100, 200ug/ml). Western blot analyses (**A**, **B**) and quantitative data (**C**, **D**) show that soluble Klotho inhibited β-catenin activation and its target gene expression induced by CTS. Results were obtained via the expression of three individual experiments performed in triplicate for each condition. *P < 0.05, **P < 0.01.
